# Effects of Automatic Deep-Learning-Based Lung Analysis on Quantification of Interstitial Lung Disease: Correlation with Pulmonary Function Test Results and Prognosis

**DOI:** 10.3390/diagnostics12123038

**Published:** 2022-12-04

**Authors:** Ryo Aoki, Tae Iwasawa, Tomoki Saka, Tsuneo Yamashiro, Daisuke Utsunomiya, Toshihiro Misumi, Tomohisa Baba, Takashi Ogura

**Affiliations:** 1 Diagnostic Radiology, Yokohama City University Graduate School of Medicine, 3-9 Fukuura, Kanazawa-ku, Yokohama 236-0004, Kanagawa, Japan; 2 Department of Radiology, Kanagawa Cardiovascular and Respiratory Center, 6-16-1 Tomioka-higashi, Kanazawa-ku, Yokohama 236-0051, Kanagawa, Japan; 3 Kanazawa Institute of Technology, 7-1 Ohgigaoka, Nonoichi 921-8501, Ishikawa, Japan; 4 Department of Biostatistics, Yokohama City University School of Medicine, 3-9 Fukuura, Kanazawa-ku, Yokohama 236-0004, Kanagawa, Japan; 5 Department of Respiratory Medicine, Kanagawa Cardiovascular and Respiratory Center, 6-16-1 Tomioka-higashi, Kanazawa-ku, Yokohama 236-0051, Kanagawa, Japan

**Keywords:** computed tomography, deep-learning, interstitial lung disease, quantitative analyses

## Abstract

We investigated the feasibility of a new deep-learning (DL)-based lung analysis method for the evaluation of interstitial lung disease (ILD) by comparing it with evaluation using the traditional computer-aided diagnosis (CAD) system and patients’ clinical outcomes. We prospectively included 104 patients (84 with and 20 without ILD). An expert radiologist defined regions of interest in the typical areas of normal, ground-glass opacity, consolidation, consolidation with fibrosis (traction bronchiectasis), honeycombing, reticulation, traction bronchiectasis, and emphysema, and compared them with the CAD and DL-based analysis results. Next, we measured the extent of ILD lesions with the CAD and DL-based analysis and compared them. Finally, we compared the lesion extent on computed tomography (CT) images, as measured with the DL-based analysis, with pulmonary function tests results and patients’ overall survival. Pearson’s correlation analysis revealed a significant correlation between DL-based analysis and CAD results. Forced vital capacity was significantly correlated with DL-based analysis (r = 0.789, *p* < 0.001 for normal lung volume and r = −0.316, *p* = 0.001 for consolidation with fibrosis volume). Consolidation with fibrosis measured using DL-based analysis was independently associated with poor survival. The lesion extent measured using DL-based analysis showed a negative correlation with the pulmonary function test results and prognosis.

## 1. Introduction

Interstitial lung diseases (ILDs) are varied and heterogenous diseases [1]. Recently, progressive pulmonary fibrosis (PPF) has been defined in patients with radiological evidence of pulmonary fibrosis and shows at least two of the following three criteria occurring within the past year: (1) worsening respiratory symptoms, (2) physiological evidence of disease progression (absolute decline in forced vital capacity (FVC) and diffusing capacity of the lung for carbon monoxide (DLCO), and (3) radiological evidence of disease progression [2]. In this context, qualitative and quantitative analysis of computed tomography (CT) plays a growing role in identifying PPF to introduce antifibrotic drugs [3,4].

Computer-based quantification of CT images based on computer-aided diagnosis (CAD) can provide an objective and reproducible measure of the lesion extent of ILD. However, the traditional CAD system cannot differentiate consolidation with fibrosis (traction bronchiectasis) from that without fibrosis. Quantification with fully manual tracing and CAD with manual corrections are the existing solutions to this problem. Specifically, a manual tracing method by an experienced chest radiologist may be the best approach for ILD classification and volumetry. However, these existing techniques are time-consuming, and they are impractical for clinical settings [5].

Recently, artificial intelligence-based methods have been applied for CT evaluation of ILD segmentation and diagnosis [6,7,8,9]. These systems are useful methods because they can minimize the need for manual tracing or corrections. Still, these systems cannot differentiate between two distinct entities, i.e., consolidation with and without fibrosis (traction bronchiectasis). Accurate classification and quantification are important for the prognostication of ILD [10,11]. Thus, we have developed a fully automatic deep-learning (DL)-based lung analysis model that can differentiate consolidation with fibrosis, such as pleuroparenchymal fibroelastosis (PPFE)-like lesions, from consolidation without fibrosis. As the PPFE-like lesions are associated with ILD severity [12,13,14,15,16], it is crucial to distinguish and separately quantify consolidation with and without fibrosis. To our knowledge, no other DL software or CAD systems have this ability [10,17,18,19]. We hypothesized that our DL-based analysis model would provide automatic and more detailed classification and quantification of ILD.

The present study aimed to quantitatively and qualitatively evaluate the effects of DL-based analysis on ILD characterization by comparing it to the traditional quantitative measures, i.e., visual evaluation by a radiologist and evaluation using a CAD system. Furthermore, we investigated the association between the quantitative results of the DL-based analysis and the disease severity and prognosis in ILD patients.

## 2. Materials and Methods

This study consisted of two steps, i.e., (1) the development of the DL-based analysis model (pre-clinical study) and (2) a clinical study of DL-based analysis.

**Figure 1:**
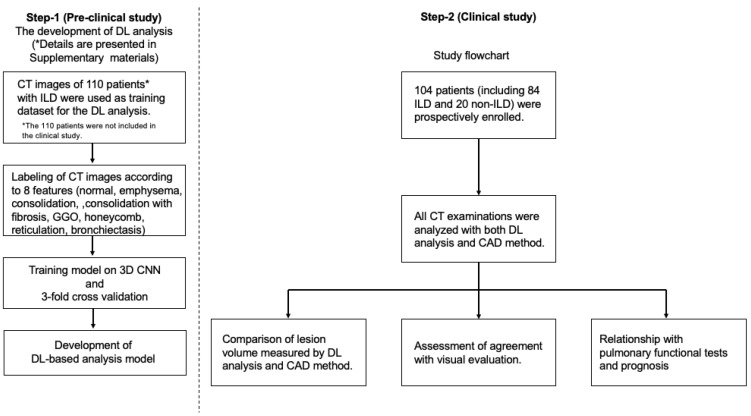
Flowchart of the study design. Step-1 (the development of deep-learning (DL)-based analysis) details described in the Supplementary Materials (*).

The study design is shown in Figure 1.

### 2.1. Patient Population

The review board of our institution approved the study protocol. Written informed consent was prospectively obtained from all the patients who underwent CT examinations to evaluate ILD or other pulmonary diseases between June 2016 and August 2018 (Figure 1).

For the step-1 preclinical study, we used CT images of 110 patients with ILD who were not included in the subsequent clinical study (Supplementary Materials) (Figure 1).

For the step-2 clinical study, we enrolled 104 patients whose characteristics are shown in Table 1.

Eighty-four ILD patients (55 men, 29 women; mean age, 69.2 ± 9.0 years) included 40 idiopathic pulmonary fibrosis (IPF) patients and 44 non-IPF ILD patients. ILD was diagnosed by multidisciplinary discussion according to the criteria established by the latest international consensus guidelines [2,20,21]. Twenty non-ILD patients (18 men and 2 women; mean age, 66.2 ± 10.9 years) were included, i.e., 12 with chronic obstructive pulmonary disease, 4 with small lung nodules (<3 cm in diameter), 2 with pleural plaques, and 2 without lung lesions.

### 2.2. CT Examination Protocol

Thin-section non-contrast CT images were obtained during inspiration with the patient in a supine position using 320- or 64-row multi-detector CT scanners (Aquilion ONE GENESIS or Aquilion 64, Canon Medical Systems, Otawara, Japan). The scanning parameters were 0.5-mm collimation, 0.5-sec gantry rotation speed, tube voltage of 120-kVp, and tube current of 250–300 mA (automatic exposure control). CT images were reconstructed with a slice thickness of 0.5 mm using an iterative reconstruction algorithm (AIDR3D; Canon Medical Systems).

### 2.3. ILD Classification with CAD and the DL-Based Algorithm

The ILD classification and lesion extent on CT images were estimated using the CAD system and DL-based analysis method.

- CAD system (Gaussian histogram normalized correlation segmentation [GHNC] estimation)

The GHNC system (Mebius Corporation, Yokohama, Japan) is a traditional CAD software that divides the lung into six categories based on the CT attenuation values and values on the differential images [22,23,24,25]. We measured the extent of the following regions: normal (N_CAD_), emphysema (E_CAD_), ground-glass opacity (G_CAD_), consolidation (C_CAD_), reticulation (R_CAD_), and honeycombing including traction bronchiectasis (H_CAD_). The total fibrotic lesions (Fib_CAD_) were calculated as the sum of R_CAD_ and H_CAD_.

- DL-based analysis (QZIP-ILD)

**Figure 2:**
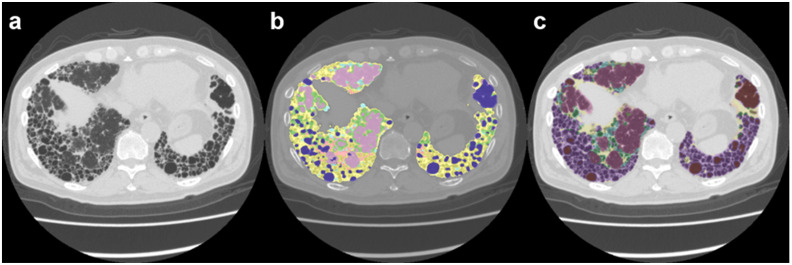
Images of a 72-year-old man with idiopathic pulmonary fibrosis (IPF). (**a**) Computed tomography image; (**b**) computer-aided diagnosis (CAD) method image; (**c**) deep-learning (DL)-based analysis image. Note: For the CAD image, purple = N_CAD_; light green = G_CAD_; pink = C_CAD_; light blue = R_CAD_; yellow = H_CAD_; and dark blue = E_CAD_. For the DL-based analysis image, violet = N_DL_; light green = G_DL_; yellow = CF_DL_; light blue = R_DL_; dark green = T_DL_; purple = H_DL_; and brown = E_DL_.

The DL-based ILD analysis system (Quantification by Ziosoft Informatics Platform for interstitial lung disease, QZIP-ILD, Ziosoft, Inc. Tokyo, Japan) is a research software, and its development details (convolutional neural network) are described in the Supplementary Materials. QZIP-ILD can classify the lung into eight features (Figure 2). We measured the extent of these features: normal (N_DL_), emphysema (E_DL_), ground-glass opacity (G_DL_), consolidation (C_DL_), consolidation with fibrosis (traction bronchiectasis) (CF_DL_), reticulation (R_DL_), honeycomb (H_DL_), and traction bronchiectasis (T_DL_). We calculated the total fibrotic lesions (Fib_DL_) as the sum of CF_DL_, R_DL_, H_DL_, and T_DL_, corresponding to Fib_CAD_.

DL-based analysis was developed to classify the lung regions into eight categories, including two more categories (consolidation with fibrosis and traction bronchiectasis) than the CAD system. The classification of the lung lesions based on the two methods is summarized in Table 2.

### 2.4. Comparison of Lung Region Volume Measured by CAD and DL-Based Method

The original number of categories of the CAD system (six categories) and DL-based analysis (eight categories) differed from each other. Therefore, we compared the volumes (mm^3^) of five different lung CT imaging feature categories (N [normal lung], E [emphysema], G [GGO], C [consolidation], and Fib [total fibrotic lesion]) measured using the CAD system and DL-based analysis. The agreement on the measured volume of each CT imaging feature category between the CAD and DL-based analysis was evaluated.

### 2.5. Accuracy for ILD Classification by CAD and DL-Based Method

One expert radiologist defined a total of 549 regions of interest (ROIs) on the CT images of the 104 patients (3 to 7 ROIs per patient), including the region of normal, emphysema, GGO, consolidation, consolidation with fibrosis, reticulation, honeycomb, and traction bronchiectasis. We chose a 3-mm diameter ROI because the size was sufficiently large to avoid missing characteristic imaging features and not so large that it would be affected by other structures surrounding the ROI. We measured the proportion of pixel numbers for each ROI and calculated the accuracy. We also evaluated the relationship between the segmentation results of the DL-based analysis and CAD methods, which were displayed as a chord diagram using the Power BI analysis tool (Microsoft Corporation, Redmond, Washington, WA, USA).

### 2.6. Relationship of DL Lung Analysis, Pulmonary Functional Tests, and Patients’ Prognosis

We compared the lung volumes of the N_DL_, G_DL_, C_DL_, CF_DL_, R_DL_, H_DL_, T_DL_, E_DL,_ and Fib_DL_ (CF_DL_+R_DL_ + H_DL_ + T_DL_) categories and that of the whole lung volume, as measured with DL-based analysis, with the pulmonary function test results (i.e., FVC, diffusing capacity of the lung for the carbon monoxide (DLCO), forced expiratory volume in 1 s (FEV1), and total lung capacity (TLC)).

We compared the DL-based analysis parameters and characteristics (age and sex) of the 104 patients with survival time.

### 2.7. Statistical Analysis

SPSS Statistics version 28 (IBM, Armonk, NY, USA) was used for the data analysis. All numerical data are reported as mean ± standard deviation. Bland-Altman plots were used to evaluate the agreement of lesion volumes between the CAD system and DL-based analysis. We also used Pearson’s correlation coefficients to compare the lesion volumes as measured with the CAD and DL-based methods. Survival time was analyzed from the date CT scanning was performed to the date of death or censoring. Univariate and multivariate Cox regression analyses were used to determine variables related to survival. A *p*-value < 0.05 was considered statistically significant.

## 3. Results

### 3.1. ILD Classification, Volumetry, and Accuracy by CAD and DL-Based Method

All CT data were successfully segmented using both the CAD system and DL-based analysis. Representative images are shown in Figure 2.

**Figure 3:**
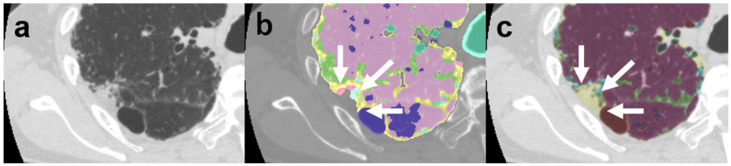
Images of a 72-year-old man with idiopathic pulmonary fibrosis. (**a**) Computed tomography image; (**b**) computer-aided diagnosis (CAD) method image; (**c**) deep-learning (DL)-based image. On the CAD method image (**b**), the pleuroparenchymal fibroelastosis (PPFE)-like lesion in the peripheral area (white arrow) was neglected because it was considered extrapulmonary. On the DL-based analysis image (**c**), the PPFE-like lesion (white arrow) is accurately classified as consolidation (CF_DL_). The color code follows that described in the legend of Figure 2.

A manual operation was not required with the DL-based analysis because the system was designed to detect lung contours and classify and measure lesions automatically; the CAD system required a manual operation to detect the lung contours in all patients. The mean time required to prepare for and complete the manual operation of the CAD lung analysis (tracing lung contours) was 17.3 ± 3.9 min per case. In some patients, consolidation with or without traction bronchiectasis in the peripheral zone was missed owing to manual correction errors (Figure 3).

**Figure 4:**
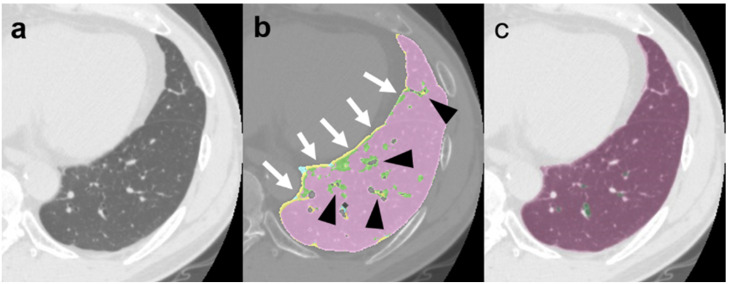
Images of a 57-year-old man with normal lungs. (**a**) Computed tomography (CT) image; (**b**) computer-aided diagnosis (CAD) method image; (**c**) deep-learning (DL)-based image. The conventional CT image (**a**) shows a normal lung. On the CAD method image (**b**), the pulmonary surface (white arrow) and perivascular area (black arrowhead) were misclassified as ground-glass opacity (G_CAD_) and honeycombing (H_CAD_), respectively. The DL-based analysis image (**c**) accurately classifies the normal lung area. The color code follows that described in the legend of Figure 2.

Additionally, in the CAD system, the peripheral vessels and heart contours were misclassified as GGO (G_CAD_) and honeycombing (H_CAD_). Some perivascular areas were misjudged as GGO (Figure 4).

**Figure 5:**
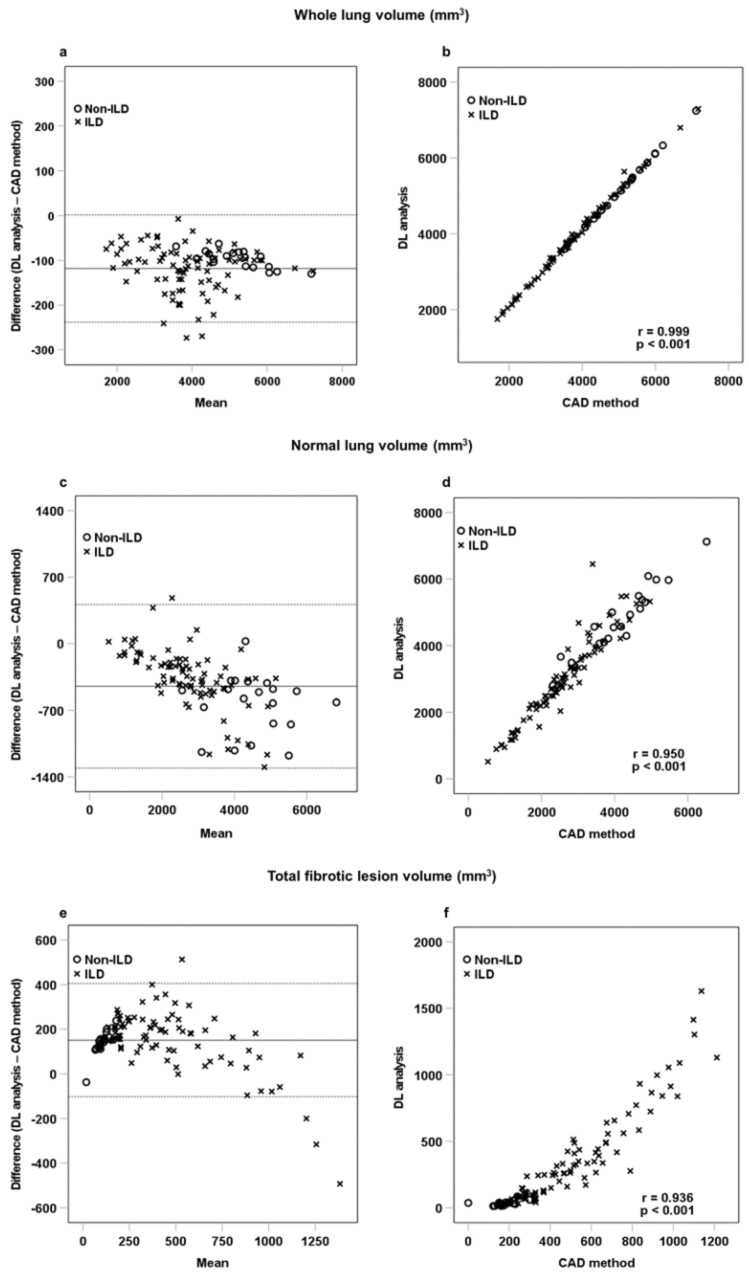
Bland-Altman plots and linear correlation of whole lung volume (**a**,**b**), normal lung volume (**c**,**d**), and fibrotic lesion volume (**e**,**f**), as determined with the CAD and DL-based methods. The black horizontal line represents the mean difference between CAD method and DL-based analysis. The dotted horizontal lines represent ±1.96 standard deviation. The difference indicates the volume as measured with the CAD method minus the volume as measured with the DL-based method.

Table 3 shows a comparison of volume between the CAD system and DL-based analysis. Bland-Altman analysis and Pearson correlation testing revealed a good correlation between whole lung, normal lung, GGO, consolidation, total fibrotic lesion, and emphysema measures. Figure 5 shows the Bland-Altman plots and the linear correlation results between whole lung, normal lung, and fibrotic lesion volumes obtained with the CAD system and DL-based analysis. Furthermore, the DL-based analysis revealed significantly higher whole lung (118.3 ± 61.1 mm^3^, *p* < 0.001), normal lung (447.8 ± 438.6 mm^3^, *p* < 0.001), and consolidation (40.0 ± 42.6 mm^3^, *p* < 0.001) volumes. Conversely, the calculated volume of total fibrotic lesions was significantly lower (−151.5 ± 129.5 mm^3^, *p* < 0.001) with the DL-based analysis than with the CAD method.

**Figure 6:**
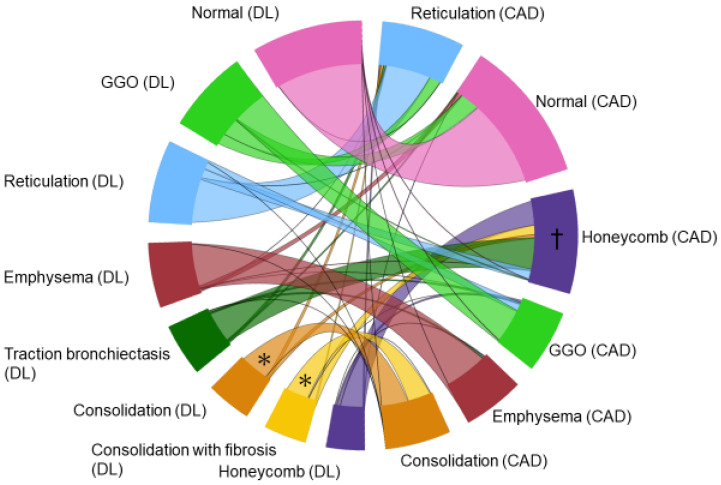
Chord diagram comparing the CAD and DL-based method outputs. The color of the chord is set to match the DL-based analysis. Most pixels that the CAD system interpreted as consolidation, the DL-based method divided into consolidation with and without fibrosis (asterisk). Most pixels that the CAD system interpreted as honeycombing (dagger), the DL-based method divided into honeycombing, traction bronchiectasis, reticulation, and consolidation with fibrosis.

One expert radiologist defined 549 ROIs on 104 CT scans, including normal, emphysema, consolidation, fibrosis, GGO, honeycomb, reticulation, and traction bronchiectasis, and compared the output results using DL-based analysis and the CAD system. The accuracy for each lesion and for the normal lung area according to the DL-based analysis and CAD system was 0.922–1.000 and 0.529–0.995, respectively (Table 4). The relationship between the segmentation results derived from DL-based analysis and the CAD system is displayed on a chord diagram (Figure 6), indicating that most pixels interpreted to reflect normal and emphysema tissue with the DL-based method were also interpreted to reflect normal and emphysema tissue with the CAD method. Conversely, what the CAD system interpreted as consolidation, the DL-based method divided into consolidation with and without fibrosis. Similarly, what the CAD system interpreted as honeycombing, the DL-based method divided into honeycombing, traction bronchiectasis, and consolidation with fibrosis.

### 3.2. Correlation with Pulmonary Function and Prognosis

Table 5 shows the correlations between the DL-based analysis results and pulmonary function test results. The median time between the CT and pulmonary function test dates was 7.5 days. The normal lung and whole lung volumes on DL-based analysis results were significantly correlated with all lung function test results (TLC, FVC, FEV1, and DLCO). Furthermore, CF_DL_ volume was inversely correlated with TLC (r = −0.224, *p* = 0.039), FVC (r = −0.316, *p* = 0.001), and DLCO (r = −0.399, *p* < 0.001).

Of the 104 patients, 16 died during the study period. The median follow-up time was 3.3 years. Univariate and multivariate Cox regression analyses were performed to identify the prognostic factors. Table S3 shows the univariate Cox regression analysis of all DL-based analysis and CAD system lesion parameters concerning survival. Table S4 shows the univariate and multivariate cox regression model with age, sex, and CF_DL_. Univariate analysis showed that age (hazard ratio [HR] = 1.096, 95% confidence interval [CI] =1.023–1.173, *p* = 0.009) and CF_DL_ (HR = 1.507, 95% CI = 1.310–1.733, *p* < 0.001) were statistically significant, while sex (HR = 1.857, 95% CI = 0.529–6.524, *p* = 0.334) was not statistically significant. Multivariable analysis, including age and CF_DL,_ revealed that age (HR = 1.091, 95% CI = 1.007–1.183, *p* = 0.034) and CF_DL_ (HR = 1.477, 95% CI = 1.277–1.708, *p* < 0.001) were independently associated with shorter survival.

## 4. Discussion

In the present study, DL-based analysis appropriately classified typical ILD lesions, in agreement with the classification provided by the expert chest radiologist. The DL-based analysis allows for automatic measurement, rendering it free from operator-dependent errors and variability, which is clinically helpful. Furthermore, DL-based analysis can quantify consolidation by distinguishing between the presence and absence of bronchiectasis. The lesions segmented into consolidation by CAD system were segmented into two patterns by DL-based analysis: consolidation without bronchiectasis (C_DL_) and consolidation with bronchiectasis (CF_DL_). The lesion extent measured with DL-based analysis correlated well with FVC, and multivariate Cox regression analysis showed that CF_DL_ was independently associated with a worse prognosis. We consider consolidation with bronchiectasis, i.e., CF_DL_, a critical index correlating with patients’ prognosis in ILD.

The previous study regarding DL-based technique for the ILD identification also reported excellent classification of ILD [10]. Our study showed similar results, but our DL-based analysis developed for this study is distinguished by its ability to quantify consolidation with traction bronchiectasis, and this is a unique capability that has not been reported in any other system. Furthermore, we revealed the quantitative results were well correlated with the clinical disease severity and prognosis. We believe our DL-based model which can identify and quantify the ILD imaging features, particularly the consolidation with fibrosis, may be useful for ILD-patient management. We believe that the ability of the DL-based method to differentiate “consolidation with fibrosis,” which is consolidation with traction bronchiectasis, comprises a unique strength, not shared with other software or CAD systems [10,17,18,19]. Consolidation without bronchiectasis is a representative finding of cryptogenic organizing pneumonia, a type of interstitial pneumonia with a good prognosis [20]. On the other hand, consolidation with traction bronchiectasis in PPF has a poor prognosis, according to previous studies [12,13,14,15]. It has also been reported that consolidation with traction bronchiectasis in the middle lobe is associated with poor prognosis in patients with an anti-aminoacyl tRNA synthetase antibody-associated ILD [26]. Our study results showed that consolidation with traction bronchiectasis was associated with prognosis. We believe that differentiating consolidation by the presence or absence of bronchiectasis may be helpful in patient management, e.g., for the prediction of the response to treatment.

The ability to automatically quantify these lesions is another advantage of the DL-based analysis. The CAD system requires the manual correction of lung contours, especially in the case of consolidation with and without fibrosis. These manual corrections might lead to estimation errors. We believe that this may be one of the reasons for the smaller consolidation extent observed with the CAD system than with the DL-based analysis. Furthermore, it has been reported that the CAD system misinterprets some peripheral structures as fibrotic lesions [24]; this may be one of the reasons for the greater volumes of total fibrotic lesions obtained with the CAD system than with the DL-based analysis. DL-based analysis is also advantageous for the more precise classification and measurement of ILD lesions over the CAD system.

We believe that DL-based analysis is useful for more than evaluation of ILD. For example, DL-based analysis can be useful in assessing the severity of infectious pneumonia, such as coronavirus disease 2019 (COVID-19) [27,28]. We also think our DL-based analysis is a potentially useful tool for the health care screening of lung cancer. It is known that both severe and mild ILD (e.g., interstitial lung abnormalities) is associated with mortality and lung cancer incidence [29]. Quantitative evaluations of the screening CT might detect a lung tumor as increased volume of consolidation or GGO. Future studies should be conducted to verify the value of DL analysis model for the serial low-dose lung-screening CT [30,31].

The present study had several limitations. First, the results were obtained from a small number of patients recruited from a single center, and our study lacked a power analysis. This was not a case-control study, but a feasibility study of new analytic technique, i.e., a DL-based analysis model for the quantitative and qualitative evaluation of ILD. In addition, the number of cases available for this study was limited because it was a single-center prospective study and because a large number of cases had to be used as training data for the DL analysis development. In addition, we did not evaluate CT images from scanners of different vendors or those with various reconstruction methods. Furthermore, the DL-based analysis developed for this study is not currently capable of detecting pulmonary nodules. As the detection of lung cancer in patients with ILD is important for each patient’s prognosis, the system may need to be used in combination with other artificial intelligence-based methods for detecting pulmonary nodules. Despite these limitations, we believe that our study results demonstrate that the DL-based analysis is clinically appropriate for the evaluation of ILD. The usefulness of the DL-based analysis presented here should be verified in greater detail in future studies.

In conclusion and recommendation, QZIP-ILD, our newly developed DL-based analysis model, can accurately and automatically detect, characterize, and quantify the extent of ILD. The DL-based analysis can distinguish between consolidation with and without fibrosis, which could not be achieved by previously reported systems. The quantitative results of DL-based analysis correlated with pulmonary function tests, and consolidation with fibrosis was associated with patient prognosis. We regard ILD evaluation with the newly developed DL-based analysis model as clinically applicable, leading to the appropriate management of ILD patients.

### The Training Process of DL-Based Analysis Model (Pre-Clinical Study)

The following steps were taken to develop the DL-based analysis model (QZIP-ILD, Ziosoft) (Figure 1). The first step in constructing the model was to create a dataset. The dataset consisted of 110 patients with ILD who were not included in the clinical study. Several cross-sections were selected, one or more from each of the three cross-sections (axial, coronal, and sagittal) of each patient, and within those cross-sections, eight features of ILD (normal, emphysema, consolidation, consolidation with fibrosis, GGO, honeycomb, reticulation, and traction bronchiectasis) were labeled on these cross-sections. The average number of cross-sections per patient was approximately 24, and the average area labeled per patient was 236,341 mm^2^. Labeling was performed by a radiologist with 30 years of experience in chest imaging.

Next, we used this dataset for training. The training model was based on a three-dimensional (3D) convolutional neural network for semantic segmentation and had a structure similar to that of a 3D U-Net, as shown in Figure S1. The network consisted of a contracting path in the first half and an expansive path in the second half, as in U-Net. Both networks were based on a convolution block consisting of a 3 × 3 × 3 convolution, an activation layer, and batch normalization. The contracting path was constructed using a convolution block and pooling. The expansive path was built with a cascade of concatenations of the up-sampled feature and the output of the contracting path and passing it through the convolution block. The CT data were resampled at 0.65 mm, the input was cropped blocks of 96 × 96 × 96 voxels, and the output was composed of eight labels corresponding to each position. The 96 × 96 × 96 blocks were randomly sampled to ensure that a block included some of the labeled regions. The model was optimized using Adam (learning rate: 0.001, betas: (0.9, 0.999)) with the cross-entropy function as the loss function.

For training, the dataset was divided into three groups such that the eight labels were evenly distributed. The total areas of the labels in each group are listed in Table S1. Using these three groups, we performed a three-fold cross-validation as shown in the solid line in Figure S2. The performance of the individual model is presented in Table S2. The final model was an ensemble of the outputs of the three models (dotted line in Figure S2).

## Figures and Tables

**Table 1:** Patient characteristics in clinical study.

Demographics and Clinical Characteristics			Number (%), or Mean (SD)
Total Number of Patients			104
Age (years)			69 (9.5)
Sex (M/F)			73/31 (70.2/29.8%)
Clinical diagnosis	ILD	IPF	40 (38.5%)
		CTD-ILD	18 (17.3%)
		Unclassifiable idiopathic interstitial pneumonia	16 (15.4%)
		Nonspecific interstitial pneumonia	3 (2.9%)
		Chronic hypersensitivity pneumonia	4 (3.8%)
		Organizing pneumonia	1 (1.0%)
		Idiopathic pleuroparenchymal fibroelastosis	2 (1.9%)
	Non-ILD	COPD	12 (11.5%)
		Pulmonary nodules	4 (3.8%)
		Pleural plaques	2 (1.9%)
		No lesion	2 (1.9%)
Follow-up period (days)			1035 (490.2)

IPF, idiopathic pulmonary fibrosis; CTD, connective tissue disease; ILD, interstitial lung disease; COPD, chronic obstructive pulmonary disease; SD, standard deviation.

**Table 2:** Classification of the lung lesions according to the CAD and DL-based methods.

	Classification	
Lesion characteristics	CAD (GHNC)	DL (QZIP)
Normal	N_CAD_	N_DL_
Emphysema	E_CAD_	E_DL_
Ground glass opacity	G_CAD_	G_DL_
Consolidation	C_CAD_	C_DL_
Consolidation with fibrosis (traction bronchiectasis)	-	CF_DL_
Reticulation	R_CAD_	R_DL_
Honeycombing	H_CAD_	H_DL_
Traction bronchiectasis	-	T_DL_
Total fibrotic lesion	Fib_CAD_ = R_CAD_ + H_CAD_	Fib_DL_ = CF_DL_ + R_DL_ + H_DL_ + T_DL_

CAD: computer-aided diagnosis, DL: deep learning.

**Table 3:** Descriptive Outcomes and Method-Comparison Analysis for the Quantification of CT Images using the CAD system versus DL-based analysis.

Lesion Pattern	Volume (mm^3^)		Paired-Samples *t*-Test	Pearson Correlation (r, *p*-Value)	Bland-Altman Analysis (Bias [95%CI])
	CAD System (Mean [SD])	DL-Based Analysis (Mean [SD])			
Whole lung	4059.8 (1180.1)	4178.2 (1180.0)	*p* < 0.001	r = 0.999, *p* < 0.001	−118.3 (−130.2 to −106.4)
Normal lung	N_CAD_: 2883.8 (1158.3)	N_DL_: 3331.5 (1398.1)	*p* < 0.001	r = 0.950, *p* < 0.001	−447.8 (−533.0 to −362.5)
GGO	G_CAD_: 278.6 (131.7)	G_DL_: 197.0 (203.7)	*p* < 0.001	r = 0.719, *p* < 0.001	81.6 (53.9 to 109.3)
Consolidation	C_CAD_: 14.7 (35.5)	C_DL_: 54.7 (61.7)	*p* < 0.001	r = 0.742, *p* < 0.001	−40.0 (−48.3 to −31.7)
Total fibrotic lesion	H_CAD_: 337.5 (205.7)	CF_DL_: 39.8 (64.1)	*p* < 0.001	r = 0.936, *p* < 0.001	151.5 (126.3 to 176.7)
		H_DL_: 71.4 (161.4)			
	R_CAD_: 131.0 (128.0)	R_DL_: 152.1 (156.9)			
		T_DL_: 53.9 (58.1)			
Emphysema	E_CAD_: 414.3 (647.0)	E_DL_: 55.8 (130.0)	*p* < 0.001	r = 0.795, *p* < 0.001	136.4 (59.7 to 213.1)

CAD: computer-aided diagnosis, CI: confidence interval, CT: computed tomography, DL: deep learning, GGO: ground-glass opacity, SD: standard deviation.

**Table 4:** DL-based analysis and CAD system output accuracy of each feature category.

	Number of ROIs	DL-Based Analysis Accuracy (95% Confidence Interval)	CAD Accuracy (95% Confidence Interval)
Normal	108	1.00	0.995
		(1.00–0.989)	(1.00–0.989)
Emphysema	66	0.995	0.887
		(1.00–0.988)	(0.941–0.831)
Ground-glass opacities	85	0.922	0.529
		(0.986–0.959)	(0.600–0.446)
Consolidation	63	0.995	0.803
		(1.00–0.980)	(0.762–0.563)
Consolidation with fibrosis	48	0.973	-
		(0.993–0.958)	
Honeycomb	39	0.976	0.792
		(0.996–0.957)	(0.857–0.727)
Reticulation	82	0.984	0.706
		(0.995–0.975)	(0.771–0.639)
Traction bronchiectasis	58	0.943	-
		(0.963–0.915)	

CAD: computer-aided diagnosis, DL: deep learning, ROI: region of interest.

**Table 5:** Correlations between the DL-based analysis results and pulmonary function test results.

Pulmonary Function Test	N	Categories in the DL-Based Analysis									
		Whole Lung	N_DL_	G_DL_	C_DL_	CF_DL_	H_DL_	R_DL_	T_DL_	E_DL_	Fib_DL_
TLC	85	*r* = 0.924	*r* = 0.836	*r* = −0.234	*r* = −0.321	*r* = −0.224	*r* = −0.145	*r* = −0.369	*r* = −0.246	*r* = 0.301	*r* = −0.323
		*p* < 0.001	*p* < 0.001	*p* = 0.031	*p* = 0.003	*p* = 0.039	*p* = 0.186	*p* = 0.001	*p* = 0.023	*p* = 0.005	*p* 0.003
FVC	100	*r* = 0.832	*r* = 0.789	*r* = −0.306	*r* = −0.291	*r* = −0.316	*r* = −0.073	*r* = −0.338	*r* = −0.233	*r* = 0.134	*r* = −0.288
		*p* < 0.001	*p* < 0.001	*p* = 0.002	*p* = 0.003	*p* = 0.001	*p* = 0.470	*p* = 0.001	*p* = 0.002	*p* = 0.185	*p* 0.004
FEV1	100	*r* = 0.682	*r* = 0.701	*r* = −0.158	*r* = −0.148	*r* = −0.191	*r* = −0.089	*r* = −0.199	*r* = −0.114	*r* = −0.102	*r* = −0.188
		*p* < 0.001	*p* < 0.001	*p* = 0.117	*p* = 0.142	*p* = 0.057	*p* = 0.379	*p* = 0.048	*p* = 0.257	*p* 0.314	*p* 0.061
DLCO	85	*r* = 0.617	*r* = 0.794	*r* = −0.204	*r* = −0.206	*r* = −0.399	*r* = −0.440	*r* = −0.491	*r* = −0.463	*r* = −0.130	*r* = −0.597
		*p* < 0.001	*p* < 0.001	*p* = 0.061	*p* = 0.059	*p* < 0.001	*p* < 0.001	*p* < 0.001	*p* < 0.001	*p* 0.235	*p* < 0.001

DLCO = diffusing capacity of the lung for carbon monoxide; FEV1 = forced expiratory volume in one second; FVC = forced vital capacity; ILD = interstitial lung disease; Fib_DL_= F_DL_ + H_DL_ + R_DL_ + T_DL_; TLC = total lung capacity; r = Pearson’s correlation coefficient.

## Data Availability

The data will be shared upon reasonable request to the corresponding author.

## Supplementary Materials

The following supporting information can be downloaded at: https://www.mdpi.com/article/10.3390/diagnostics12123038/s1, Table S1: Total area (mm^2^) of the labels in three group; Table S2. Performance of 3-fold cross-validation; Table S3. Univariate Cox regression analysis of survival; Table S4. Univariate and Multivariate Cox regression model analysis of survival; Figure S1. The network architecture for lesion classification; Figure S2. Training process of deep-learning and prediction process by the trained deep-learning models.
