# Supplementary material for: Effects of Automatic Deep-Learning-Based Lung Analysis on Quantification of Interstitial Lung Disease: Correlation with Pulmonary Function Test Results and Prognosis
Source: Diagnostics (Basel). 2022 Dec 4;12(12):3038. doi: 10.3390/diagnostics12123038 (PMC9777463; doi:10.3390/diagnostics12123038)
Supplement: Supplementary file 1 [file diagnostics-12-03038-s001.zip › diagnostics-2058216-supplementary.pdf]

Supplementary Files

Table S1. Total area (mm^2) of the labels in the three groups

	Number of Patients	Emphysema	Consolidation	Consolidation with fibrosis	GGO	Honeycomb	Reticulation	Traction Bronchiectasis	Normal	Total
Group 1	37	297,497.8	280,302.3	204,216.7	869,541.9	187,958.2	660,637.5	292,216.9	5,940,808.6	8,733,180.0
Group 2	37	287,187.0	272,226.5	208,950.6	860,152.5	192,823.1	624,635.8	296,019.8	5,902,124.4	8,644,119.6
Group 3	36	288,061.6	297,892.6	193,561.4	872,437.7	178,667.5	651,563.6	277,716.7	5,860,293.2	8,620,194.5
All	110	872,746.4	850,421.4	606,728.8	2,602,132.1	559,448.8	1,936,836.9	865,953.5	17,703,226.2	25,997,494.1

GGO = ground-glass opacity

Table S2. Performance of 3-fold cross-validation

		Fold 1(train 2, 3, validation 1)			fold 2(train 1, 3, validation 2)			fold 3 (train 1, 2, validation 3)		
Train		Recall	Precision	F1	Recall	Precision	F1	Recall	Precision	F1
	Emphysema	91.22%	76.47%	83.19%	90.18%	70.17%	78.92%	94.02%	74.92%	83.39%
	Consolidation	77.13%	83.99%	80.41%	82.60%	80.14%	81.35%	85.17%	82.35%	83.74%
	Consolidation with fibrosis	61.77%	76.30%	68.27%	69.69%	66.91%	68.27%	73.73%	69.36%	71.48%
	GGO	74.15%	74.92%	74.54%	72.48%	77.08%	74.71%	76.21%	76.59%	76.40%
	Honeycomb	66.39%	75.93%	70.84%	69.10%	71.52%	70.29%	83.68%	75.41%	79.33%
	Reticulation	86.36%	69.18%	76.82%	81.79%	73.27%	77.29%	80.23%	77.10%	78.63%

	Traction bronchiectasis	62.32%	67.87%	64.98%	61.83%	70.21%	65.75%	65.79%	77.49%	71.16%
	Normal	95.31%	96.91%	96.10%	95.19%	96.54%	95.86%	95.35%	96.91%	96.12%
	Macro F1			76.89%			76.56%			80.03%
Validation		Recall	Precision	F1	Recall	Precision	F1	Recall	Precision	F1
	Emphysema	91.91%	80.22%	85.67%	93.01%	73.51%	82.12%	90.55%	69.12%	78.40%
	Consolidation	79.86%	85.87%	82.76%	84.49%	81.75%	83.10%	80.05%	81.22%	80.63%
	Consolidation with fibrosis	64.60%	75.08%	69.45%	72.80%	71.60%	72.20%	68.13%	66.44%	67.28%
	GGO	78.39%	74.84%	76.57%	71.96%	76.77%	74.29%	70.67%	76.45%	73.44%
	Honeycomb	78.18%	77.89%	78.04%	71.43%	83.14%	76.84%	76.22%	59.03%	66.53%
	Reticulation	85.76%	72.67%	78.68%	84.80%	72.90%	78.40%	75.86%	74.00%	74.91%
	Traction bronchiectasis	70.88%	74.53%	72.66%	65.59%	71.81%	68.56%	54.35%	67.58%	60.25%
	Normal	94.98%	97.26%	96.11%	95.15%	96.63%	95.89%	95.73%	96.33%	96.02%
	Macro F1			79.99%			78.92%			74.68%

GGO = ground-glass opacity

Table S3. Univariate Cox regression analysis of survival

Parameters	Hazard ratio	95% CI	P
C_{DL} ratio (%)	1.291	1.084-1.538	0.004
G_{DL} ratio (%)	1.03	0.976-1.087	0.287
CF_{DL} ratio (%)	1.507	1.310-1.733	<.001
H_{DL} ratio (%)	1.156	1.072-1.248	<.001
R_{DL} ratio (%)	1.166	1.080-1.259	<.001
T_{DL} ratio (%)	1.522	1.308-1.770	<.001
E_{DL} ratio (%)	1.003	0.957-1.052	0.893
Fib_{DL} ratio (%)	1.124	1.078-1.171	<.001
C_{CAD} ratio (%)	1.709	1.345-2.171	<.001
G_{CAD} ratio (%)	1.133	1.027-1.250	0.013
H_{CAD} ratio (%)	1.183	1.118-1.252	<.001
R_{CAD} ratio (%)	1.13	1.046-1.22	0.002
E_{CAD} ratio (%)	0.984	0.937-1.034	0.522
Fib_{CAD} ratio (%)	1.168	1.102-1.237	<.001

Table S4. Univariate and multivariate Cox regression model analysis of survival

	Univariate analysis			Multivariate analysis		
Variables	HR	95%CI	P value	HR	95%CI	P value
Age	1.096	1.023-1.173	0.009	1.091	1.007-1.183	0.034
Sex	1.857	0.529-6.524	0.334	NA		
CF_{DL} ratio (%)	1.507	1.310-1.733	<0.001	1.477	1.277-1.708	<0.001

	Univariate analysis			Multivariate analysis		
Variables	HR	95%CI	P value	HR	95%CI	P value
Age	1.096	1.023-1.173	0.009	1.091	1.007-1.183	0.034
Sex	1.857	0.529-6.524	0.334	NA		
CF_{DL} ratio (%)	1.507	1.310-1.733	<0.001	1.477	1.277-1.708	<0.001

HR= hazard ratio; CI= confidence interval; NA= not considered in the multivariable model.

Figures

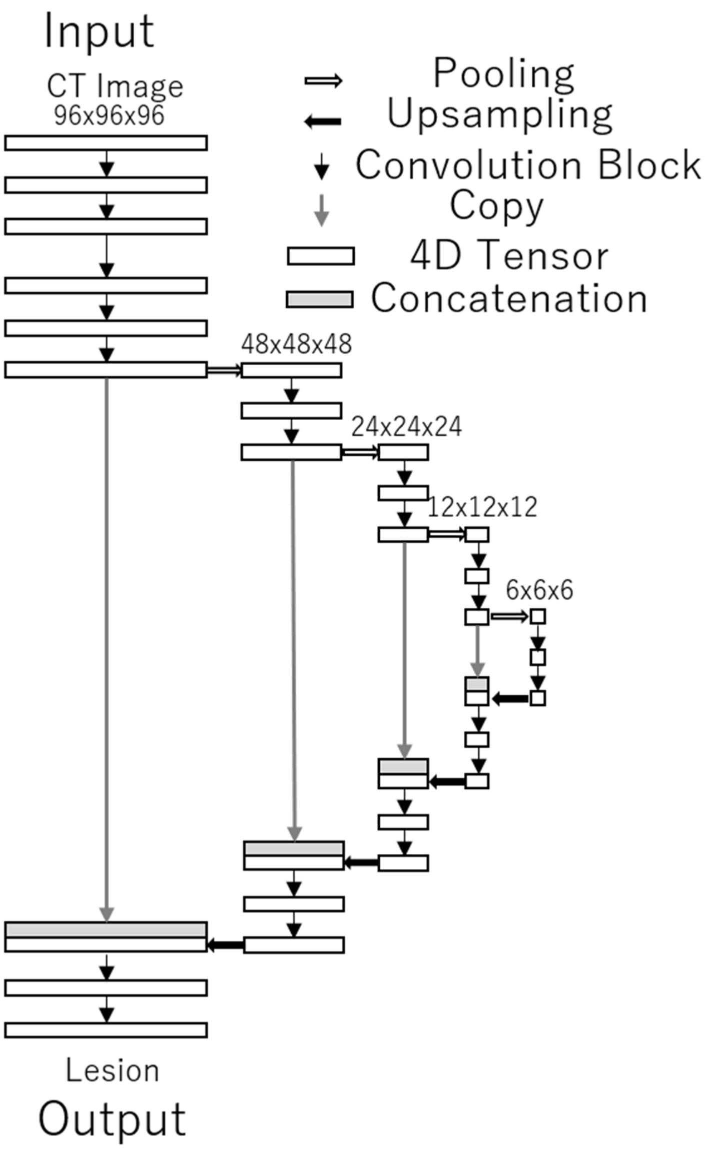

Figure S1. Network architecture for lesion classification. Each white box corresponds to the feature map. The x-y-z size is provided at the left edge, and arrows indicate each operation.

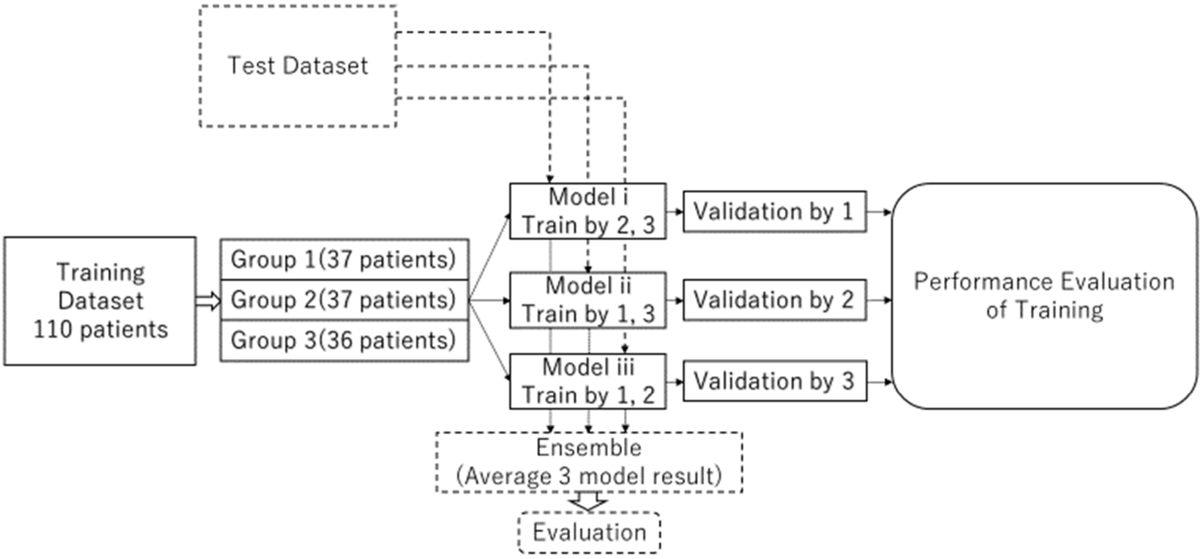

Figure S2. Training process of deep-learning (sold line) and prediction process by the trained deep-learning models (dotted line).
